# Prevalence of Diabetic Retinopathy Among Diabetic Patients from Northeastern Bulgaria

**DOI:** 10.3390/diagnostics14202340

**Published:** 2024-10-21

**Authors:** Zornitsa Zlatarova, Elitsa Hristova, Kristina Bliznakova, Virginia Atanasova, Zhaneta Yaneva, Darina Koseva, Lidiya Zaduryan, Gabriela Vasileva, Daliya Stefanova, Klara Dokova

**Affiliations:** 1 Department of Optometry and Occupational Diseases, Medical University of Varna, 9002 Varna, Bulgaria; elitsa.hristova@mu-varna.bg (E.H.);; 2 University Specialized Eye Hospital, 9002 Varna, Bulgaria; 3 Department of Medical Equipment, Electronic and Information Technologies in Healthcare, Medical University of Varna, 9002 Varna, Bulgaria; kristina.bliznakova@mu-varna.bg; 4 Department of Social Medicine and Healthcare Organization, Medical University of Varna, 9002 Varna, Bulgaria; 5 Second Department of Internal Diseases, Sector Endocrinology and Metabolic Diseases, Medical University of Varna, 9002 Varna, Bulgaria

**Keywords:** diabetic retinopathy, prevalence, Bulgaria

## Abstract

Background: Diabetic retinopathy (DR) is a leading cause of visual impairment globally among working-aged individuals. This study aims to update data on DR prevalence in Bulgaria. Methods: The present cross-sectional study was conducted between 1 January 2022 and 1 January 2023, using a local diabetes registry from the city of Varna as a sampling framework. In total, 587 diabetic patients underwent DR examination. Data included demographics, diabetes type/duration, treatment, and ophthalmic history. DR status was assessed using indirect slit-lamp biomicroscopy or digital fundus photography, graded by the International Clinical Diabetic Retinopathy Scale. Results: Of 587 participants, 13 were excluded due to cataract-related ungradable images. The median age was 65 years (IQR 56–73), with a slight female predominance (54%). The overall prevalence of any DR was 39.9% (95% CI 35.9–44.0), with non-proliferative DR (NPDR) at 27.5%, proliferative DR (PDR) at 7.3%, and macular edema (DME) at 5%. Type 1 diabetes patients had significantly higher DR prevalence (68.8%) than type 2 (34.1%, *p* < 0.001). Men exhibited higher DR prevalence. Age and diabetes duration correlated positively with DR prevalence. Insulin treatment was associated with higher DR prevalence (55.6%) than oral antidiabetic treatment (22.5%, *p* < 0.001) for type 2 diabetes patients. Among those diagnosed with DR, 70.9% received treatment, mainly laser therapy. Conclusions: These findings provide epidemiological insights for future research and emphasize the need for a comprehensive national DR screening program in Bulgaria. Technological advancements enable proactive measures to mitigate DR-related visual impairment and blindness, including widespread screening, even in rural areas.

## 1. Introduction

Diabetic retinopathy (DR) is recognized by ophthalmologists, institutions, and societies worldwide as the most important and preventable cause of visual impairment and blindness among the active population aged 20 to 74 years [1]. As the most prevalent complication of diabetes mellitus (DM), DR affects more than half of the individuals with type 1 diabetes and over one-fifth of those with type 2 diabetes [2].

The prevalence of diabetes is expected to rise in the coming decades. In Europe, 61.4 million people were living with diabetes in 2021, and this figure is expected to reach 69 million by 2045 [1]. Consequently, the number of patients affected by DR will also rise, posing a significant threat to ophthalmic health and quality of life [1].

To mitigate DR complications, clear, internationally recognized recommendations for prevention have been established, focusing on identifying at-risk individuals and implementing regular DR screening for diabetic patients [3]. Once diagnosed, individuals with DR have to be continuously monitored and offered timely ophthalmological treatment, along with management of higher levels of HbA1c, arterial pressure, and serum lipid levels, as the most effective approach to preventing severe vision damage and blindness. Information about all diabetic patients and proactive communication with them are essential for establishing effective DR preventive programs.

This explains the systematic efforts in many countries to establish diabetes and DR registries, as well as comprehensive screening programs within the frameworks of existing nationwide or regional population-based diabetic registries [4,5,6].

Bulgaria, a southeastern European country, has been a member of the European Union since 2007. It has a compulsory public health insurance system, aiming to achieve universal health coverage. However, approximately 13% of the population remains uninsured. According to the latest IDF Diabetes Atlas data, the prevalence of diabetes among adults aged 20–79 years is of 9.9% (with a 95% confidence interval of 8.3–10.6). The age-adjusted comparative diabetes prevalence for the same age group, 20–79, years is reported as 7.4% (95% CI 6.0–8.3) [1].

However, there is currently no functioning national diabetes registry in Bulgaria, resulting in limited epidemiological data on the prevalence of diabetic retinopathy [7]. The last published cross-sectional study on the epidemiology of DR was conducted 30 years ago (in 1994) in the Pirdop area, a small town with a population of around 7500 people, situated 80 km east of the capital, Sofia [8]. The authors of the Pirdop study reported a DR prevalence of 28% among a sample of 369 individuals from the diabetic population. There are no other population-based studies on this significant public health issue in Bulgaria.

Therefore, the aim of the present study is to provide more recent and adequately reported data on the prevalence of DR in a sample of diabetic patients from the city of Varna, in northeastern Bulgaria.

## 2. Materials and Methods

### 2.1. Study Design and Setting

The present study, employing a cross-sectional design, was planned and organized within the framework of a local diabetes register, covering the population 18 + years of age with diabetes, residents of Varna city. This is the third largest city in Bulgaria, located on the Black Sea coast, and has a population of 310,664 according to the 2021 census, with 250,729 individuals aged above 20 years of age [9]. The city hosts a medical university with a specialized university eye hospital, which serves the population of northeastern Bulgaria and is the sole public specialized hospital in the country. The study was conducted in the period from 1 January 2022 to 1 January 2023.

### 2.2. Eligible Target Group

The local diabetes registry included approximately 6400 registered diabetic patients aged 18 years and above. Of these, 587 underwent an eye exam during the study period.

For each participant in the study, the following information was collected and recorded: date of birth, gender, date of diabetes diagnosis, type of diabetes and type of diabetes treatment (insulin, oral, combined), and ophthalmic history.

### 2.3. Ophthalmic Examination

The ophthalmic examination assessing the DR status of the participants was conducted using one of two methods. The first method involved a detailed fundus examination with indirect slit-lamp biomicroscopy using a +90 D Volk lens, following mydriasis with 1% tropicamide. The examination was performed by an ophthalmologist specialized in retinal disease pathology. The second method involved fundus photography, conducted after mydriasis with 1% tropicamide, using a Canon CF-1 digital fundus camera. This procedure was performed by medical doctors, residents in ophthalmology, who were trained in fundus photography. Two 45° non-stereoscopic retinal digital photographs per eye were obtained, one centered on the posterior pole and the other centered on the optic disc, following the protocol applied in the English DR Screening Program and the RETINODIAB Study in Portugal [10,11]. Both eyes were evaluated for DR, and the worse grade from both eyes was used in the analysis. The readings of the images were conducted by ophthalmologists qualified in retinal pathology. Digital retinal images were considered not gradable if the retina of both eyes could not be visualized properly.

For the classification of diabetic retinopathy, the International Clinical Diabetic Retinopathy Scale was used. DR was further classified as non-proliferative (NPDR) and proliferative diabetic retinopathy (PDR). Diabetic macular edema (DME) was described separately as the presence of retinal thickening and/or hard exudates within 1 disc diameter of the center of the fovea. Diabetic retinopathy patients who had initiated treatment at the time of the examination reported what type of treatment was performed—laser therapy, intravitreal injections of anti-VEGF drugs, or pars plana vitrectomy.

### 2.4. Study Variables

The primary outcome variable was the presence or absence of diabetic retinopathy (a dichotomous variable) and the type of DR was a secondary variable. Independent variables included age (continuous), gender (dichotomous), type of diabetes (dichotomous), duration of diabetes in years (continuous), type of diabetes treatment (categorical), and type of ophthalmic treatment (categorical).

### 2.5. Ethical Consideration

The study received approval from the Ethical Committee of the Medical University of Varna (No 93/21 May 2020). All participants provided written informed consent, and the study adhered to the Declaration of Helsinki guidelines.

### 2.6. Analysis

Continuous data were presented as mean/median ± SD/IQR, and categorical data as frequencies. For normally distributed continuous data, the independent t-test was employed, while the rank sum test was used for non-normally distributed data. The χ2 test was used to test associations between categorical data. The association between diabetic retinopathy prevalence and independent variables was assessed by using a binary logistic regression analysis model. Adjusted odds ratios (AORs) were used to estimate the strength of association. All variables associated with diabetic retinopathy with a *p*-value less than 0.25 in the bivariable analysis were further analyzed using multivariable analyses. Data were analyzed using IBM SPSS version 21.0, with a significance level of 0.05 for two-sided hypothesis testing.

## 3. Results

### 3.1. Characteristics of Study Participants

A total of 587 individuals responded to the invitation for eye examination in the period 1 January 2022 to 1 January 2023. However, eye fundus examination was not possible for thirteen individuals due to the presence of cataracts.

The median age of the participants was 65 years (IQR 56–73), with women slightly more represented (54%). Individuals aged 65 years and above constituted half of the examined sample. The demographic characteristics of the participants in the study are summarized in Table 1.

Among all diabetic patients, those with type 1 diabetes accounted for 13.9% of the sample (95% CI 11.2–17.0). Study participants were categorized into three groups based on diabetes duration: less than 10 years, 10–19 years, and 20+ years duration, each representing approximately a third of the sample (Table 1).

The proportion of patients with diabetes duration exceeding 20 years was 58.4% among individuals with type 1 diabetes, significantly higher compared to those with type 2 diabetes (26.4%) (chi square = 31.171, *p* < 0.001). Patients with type 1 diabetes were significantly younger (*p* < 0.001), and had a higher proportion of men compared to the type 2 diabetes group, although there was no statistical difference in the gender distribution (*p* = 0.163) (see Table 1).

### 3.2. Diabetes Retinopathy Prevalence

The overall prevalence of any DR within the studied sample was 39.9% (95% CI 35.9–44.0), with the prevalence of NPDR at 27.5% (95% CI 23.9–31.4), PDR at 7.3% (95% 5.3–9.8), and DME at 5% (95% CI 3.4–7.2). The prevalence of DR was twice as high among patients with type 1 diabetes (68.8%) compared to those with type 2 diabetes, with DR present in more than a third of patients with type 2 diabetes (*p* < 0.001) (Table 2).

The prevalence of all forms of DR (NPDR, PDR) and DME was significantly higher among patients with type 1 diabetes as compared to the type 2 diabetes group.

Among all patients with DR (*n* = 229) in our sample, a substantial proportion, 68 (29.7%), were newly diagnosed with the disease. The overall one-year newly diagnosed DR proportion was 15.2% (95% CI12.1–18.9), with 35.0% (20.6–51.7) for diabetes type 1 and 13.3% (95% CI 10.2–17.0) for diabetes type 2 patients.

### 3.3. Diabetic Retinopathy Frequency by Participant Characteristics

Regarding the risk of newly diagnosed DR by gender, men had a higher proportion of newly diagnosed DR than women in our sample (Table 3). In terms of age, both the newly diagnosed and prevalent DR were highest in the youngest age group, 15–44 (*p* < 0.001), for patients with both type 1 and type 2 diabetes; this proportion then decreased in the subsequent age groups. The effect of diabetes duration was unidirectional, with increasing duration associated with a rise in both the newly diagnosed and prevalent DR (*p* < 0.001) (Table 3). The risk factors—gender, age, diabetes duration, and type of diabetes treatment—were included in the multiple logistic regression model. Except for gender, all others were related to the risk of DR based on the binomial and multiple logistic regression analysis.

The risk for diabetic retinopathy prevalence among the patients with type 2 diabetes was strongly associated with the type of diabetes therapy (*p* < 0.001). Those treated with insulin had a significantly higher DR prevalence (55.6%) compared to the group receiving oral antidiabetic treatment (22.5% DR prevalence).

Among the 127 individuals with known DR at the time of the examination, 90 (70.9%) reported receiving treatment for their eye disease. The largest group received treatment with laser therapy—64 patients (71% of all treated), followed by laser therapy and anti-VEGF (13 patients, 14.4%), with anti-VEGF therapy alone for 11 patients (12.2%), and 2 patients reported undergoing pars plana vitrectomy.

## 4. Discussion

This study represents the first large-scale population-based investigation into DR prevalence in a geographically well-defined area in northeastern Bulgaria. A previous report from 30 years ago provided limited data on DR epidemiology in Bulgaria, with a reported DR prevalence of 28% among 369 examined patients (1992–1994) [8]. Our findings reveal a substantially higher DR prevalence of 39.9% and of newly diagnosed DR of 15.6% per year. Additionally, our study is the first to report data on the types of DR (including newly diagnosed DR), as well as the prevalence of DME, from a cross-sectional study in Bulgaria, with an NPDR prevalence at 27.5%, PDR at 7.3%, and DME at 5.0%.

Our results, while worryingly high, are similar to DR prevalence rates reported in Dubrovnik-Neretva County, Croatia (2019) [12]. The similarities in the two studies are related to the size and the characteristics of the samples, the close geographical location (both on the Balkans), and the DR prevalence rates (39.9% in our study and 44.5% in Dubrovnik, Croatia). The prevalence of PDR was 5% in Croatia and 7.3% in Varna, Bulgaria.

Results similar to ours were reported by P. Scanlon et al. for the UK population of Gloucester, where DR prevalence declined from 38.9 (95% CI 38.1, 39.8) in 2012 to 36.6 (95% CI: 35.9, 37.3) in 2016 [13]. However, our results for DR prevalence are less favorable compared to countries with retinopathy screening traditions, such as Wales, where the overall prevalence reported was 32.4% (2005–2008) [5] or compared to the most recent data from the USA in 2021, reporting a prevalence of 26.4% [14]. Our findings also indicate a significantly lower prevalence compared to Russia, where a prevalence of 45.9% was reported for the year 2009 [15].

As observed in other studies, we also found a significant variation in DR prevalence based on the type of diabetes, with typically higher rates in type 1 diabetes. For instance, studies from Spain reported a prevalence of 36.5%, while in Norway, it was as high as 61%; in Russia, DR prevalence among individuals with type 1 diabetes was reported at 54.6% [2]. In our study, we observed a DR prevalence of 68.8% among individuals with type 1 diabetes, consistent with these findings.

Comparing our results for DR by age is challenging due to the scarcity of age-specific data. However, such data is available for the USA from the NHANES for 2021 [14]. This study reports two peaks of DR, one at the age 55–59 and the second for those aged 75–79 years. In our study, the highest DR prevalence was observed in the age group up to 44. However, when examining patients with type 2 DM, the highest prevalence was found in the age group 45–65, followed by the group 65+.

The duration of the diabetes is the other significant factor influencing DR prevalence, as indicated by a global review of DR epidemiology [2]. Our data confirm this trend, showing that patients with a diabetes duration of more than 20 years experienced a DR prevalence of 62%.

Additionally, there is an association between the type of treatment in patients with type 2 diabetes and the prevalence of DR. Our results confirm findings from other studies, indicating that the presence of DR is associated with insulin therapy in individuals with type 2 diabetes [5,12].

The proportion of newly diagnosed cases with DR in our study is unacceptably high (27.8%). In a recent German screening initiative, authors reported only 5% newly diagnosed DR patients [16].

That worrying outcome underlines the urgent need to implement a more effective approach for a national DR screening program in Bulgaria. Our results are even more worrying as the present sample was recruited from the population of a large city where access to specialist ophthalmological care is incomparably easier than in rural areas or small towns. Some guidelines recommend the integration of DR and DME screening services into clinics attended by people with diabetes [17]. However, in Bulgaria, there are no specialized diabetes centers and clinics. Care for diabetic patients is the responsibility of general practitioners, and their advice and directions for eye screening are not as efficient as a comprehensive population-based screening program for people with diabetes.

Despite the fact that 71% of treated patients with DR in this study underwent laser therapy in recent years, we registered a rapidly increasing number of patients receiving intravitreal anti-VEGF medications. Unfortunately, poor glycemic control in some diabetic patients with DME acts as a barrier to the reimbursement of this type of treatment by the National Health Insurance Company.

The strengths of the present study lie in its adherence to internationally accepted guidelines for DR screening and grading, and include the eye examinations and fundus photos assessments carried out by highly specialized experts in retinal pathology.

However, there are several limitations to consider. Firstly, the lack of a nationwide diabetes register necessitated reliance on a regional, short-term effort to register all diabetic patients in Varna City, Bulgaria. Secondly, the absence of nationwide DR screening and registration makes such regional initiatives the sole source of epidemiological information on eye complications of diabetes. A third important limitation is that the period of data collection coincided with the COVID-19 pandemic. As a result, individuals without symptoms may have avoided preventive check-ups due to concerns about potential COVID-19 infection. All three limitations are expected to influence our results in the direction of increasing the prevalence of DR. Each of these factors contributes to delayed examination, ultimately leading to higher prevalence.

## 5. Conclusions

The present study provides the most recent diabetic retinopathy prevalence data for Bulgaria. The prevalence of DR in our country is high and exceeds rates reported in developed and other central European countries.

The results emphasize the urgent need for preventive measures and the development of wide-scale population-based registration and monitoring of diabetes and all its complications, including diabetic retinopathy. With advancements in technology, DR screening is feasible and achievable not only for citizens in big cities with access to specialized ophthalmic medical care but also for those in smaller and rural areas with the appropriate use of portable fundus cameras and even smartphone-based fundus photography. Our results might also serve as important epidemiological information for future studies on the prevalence of retinopathy among diabetes patients.

## Figures and Tables

**Table 1:** Characteristics of the study participants, by diabetes type.

	Total	Diabetes Type 1	Diabetes Type 2	*p*
*n* *	574	80	494	
Age, years, mean (SD) **	63.1 (13.14)	45.7 (14.04)	65.9 (10.59)	<0.001
Diabetes duration, years, mean (SD)	14.5 (9.66)	22.1 (11.98)	13.1 (8.51)	<0.001
Gender				
Female, *n* (%)	314 (54.7)	38 (47.5)	276 (55.9)	0.163
Male, *n* (%)	260 (45.3)	42 (52.5)	218 (44.1)	
Age groups				
15–44, *n* (%)	56 (9.8)	38 (47.5)	18 (3.7)	<0.001
45–64, *n* (%)	216 (37.7)	33 (41.3)	183 (37.1)	
65–90, *n* (%)	301 (52.5)	9 (11.3)	292 (59.2)	
Years from diagnosis				
<10, *n* (%)	170 (33.7)	16 (20.8)	154 (36.0)	<0.001
10–19, *n* (%)	177 (35.0)	16 (20.8)	161 (37.6)	
≥20, *n* (%)	158 (31.3)	45 (58.4)	113 (26.4)	
Type of diabetes therapy				
Oral, *n* (%)	275 (48.0)		275 (55.8)	<0.001
Insulin, *n* (%)	206 (35.9)	80 (100.0)	126 (25.6)	
Combined, *n* (%)	42 (7.3)		42 (8.5)	

* *n*—number; ** SD—standard deviation.

**Table 2:** Diabetic retinopathy prevalence by type of diabetes.

DR * Indicator	Total	Diabetes Type 1	Diabetes Type 2	*p*
	*n* = 574	*n* = 80	*n* = 494	
Prevalence DR *n* (%)	229 (39.9)	55 (68.8)	174 (35.2)	*p* < 0.001
NPDR ** prevalence *n* (%)	158 (27.5)	40 (50.0)	118 (23.9)	*p* < 0.001
PDR *** prevalence *n* (%)	42 (7.3)	15 (18.8)	27 (5.5)	*p* < 0.001
DME ^♣^ prevalence *n* (%)	29 (5.0)	7 (8.8)	22 (4.5)	0.105
Newly diagnosed DR *n* (per 100/year)	68 (15.2)	14 (35.0)	54 (13.3)	<0.001

* DR—diabetic retinopathy; ** NPDR—non-proliferative diabetic retinopathy; *** PDR—proliferative diabetic retinopathy; ^♣^ DME—diabetic macular edema.

**Table 3:** Diabetic retinopathy prevalence by patient characteristics.

Patient Characteristics	Sample	Any DR * Prevalence	*p*	Newly Diagnosed DR	Unadjusted OR	Adjusted OR
Gender	*n*	% (CI)	0.160	% (CI)		
Men	260	42.7 (36.6–48.9)		17.8 (12.7–23.8)	1	
Women	314	37.6 (32.2–43.2)		13.3 (9.3–18.2)	0.8 (0.54–1.14)	
Age group			0.001			
15–44	56	55.4 (41.5–68.7)		33.3 (19.1–50.2)	1	1
45–64	216	45.8 (39.1–52.7)		21.3 (15.3–28.6)	0.7 (0.37–1.33)	1.3 (0.63–2.86)
65–90	301	32.9 (27.6–38.5)		8.5 (5.4–12.8)	0.3 (0.17–0.62)	0.6 (0.27–1.29)
Years from diabetes diagnosis			<0.001			
<10	170	15.3 (10.2–21.6)		8.1 (4.4–13.4)	1	1
10–19	177	38.4 (31.2–46.0)		21.0 (14.6–28.6)	3.4 (2.00–5.75)	3.6 (2.00–6.34)
≥20	158	62.0 (53.9–69.6)		27.1 (18.0–37.8)	10.2 (5.89–17.68)	8.8 (4.77–16.12)
Type of diabetes therapy			<0.001			
Oral	275	22.5 (17.7–27.9)			1	1
Insulin	205	60.5 (53.4–67.2)			6.3 (4.11–9.69)	3.8 (2.32–6.28)
Combined	42	45.2 (29.9–61.3)			3.1 (1.54–6.21)	2.1 (0.97–4.44)

* DR—diabetic retinopathy.

## Data Availability

The raw data supporting the conclusions of this article will be made available by the first or corresponding authors upon request.
